# 

**Published:** 1900-01

**Authors:** 


					JANUARY, 1900.
THE
AMERICAN JOURNAL
OF
DENTAL SCIENCE.
edited!
F. J. S. GORGAS, M. D,, D. D. S.
RICHARD GRADYr^^r^>r-?r-S^
Vol. 33.
THIRD SERIES
So. 9.
Baltimore:
SNOWDEN 6. COWMAN MFG. CO., S W. FaVette St.
LONDON:
TRUBNER A. CO., 60 Paternoster Rowk
Entered at the PoBt Office at Baltimore, Md., as Second-class Matter.
P&YKBLE IN SDVRNCEJ
PUBLISHED MONTHLY.
$2.50 P^R""OTNUM.

				

